# Supplementary material for: Transcriptomic Insights into Mechanisms of Early Seed Maturation in the Garden Pea (Pisum sativum L.)
Source: Cells. 2020 Mar 23;9(3):779. doi: 10.3390/cells9030779 (PMC7140803; doi:10.3390/cells9030779)

Zhongwan−6,25(2)

Global Summary

%DE = 0.22

genes with fdr < 0.2 = 428 (201 + / 227 −)

genes with fdr < 0.1 = 381 (183 + / 198 −)

genes with fdr < 0.05 = 369 (178 + / 191 −)

genes with fdr < 0.01 = 298 (146 + / 152 −)

genes in genesets = 0

<FC> = 0

<t−score> = 0

<p−value> = 0.01

<fdr> = 0.78

Portrait

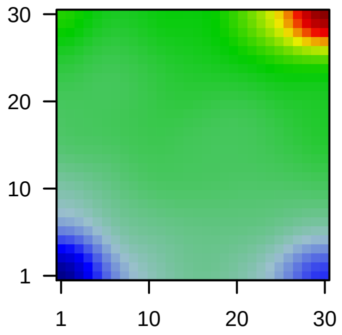

Top 100 DE genes

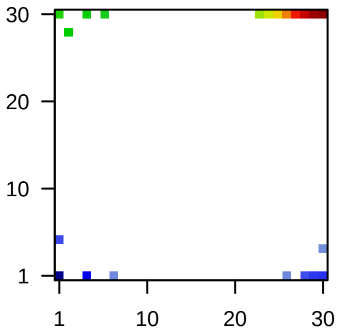

Global Genelist

Rank	ID	log(FC)	p−value	fdr	Metagene	Description
1	TRINITY_DN2	−0.53	2e−16	3e−15	1 x 1	
2	TRINITY_DN9	2.04	2e−16	3e−15	30 x 30	
3	TRINITY_DN7	−0.62	2e−16	3e−15	30 x 1	
4	TRINITY_DN7	0.58	2e−16	3e−15	30 x 30	
5	TRINITY_DN5	2.81	2e−16	3e−15	30 x 30	
6	TRINITY_DN5	0.74	2e−16	3e−15	1 x 1	
7	TRINITY_DN7	−1.28	2e−16	3e−15	1 x 1	
8	TRINITY_DN5	0.63	2e−16	3e−15	30 x 4	
9	TRINITY_DN4	−3.25	2e−16	3e−15	1 x 1	
10	TRINITY_DN9	−0.8	2e−16	3e−15	30 x 1	
11	TRINITY_DN9	0.76	2e−16	3e−15	30 x 30	
12	TRINITY_DN2	0.54	2e−16	3e−15	29 x 30	
13	TRINITY_DN2	−0.67	2e−16	3e−15	1 x 1	
14	TRINITY_DN2	−0.79	2e−16	3e−15	30 x 1	
15	TRINITY_DN2	1.1	2e−16	3e−15	30 x 30	
16	TRINITY_DN2	−0.67	2e−16	3e−15	1 x 1	
17	TRINITY_DN3	−0.62	2e−16	3e−15	28 x 1	
18	TRINITY_DN3	−1.76	2e−16	3e−15	1 x 1	
19	TRINITY_DN3	−0.57	2e−16	3e−15	1 x 1	
20	TRINITY_DN1	−1.38	2e−16	3e−15	1 x 1	

p−values

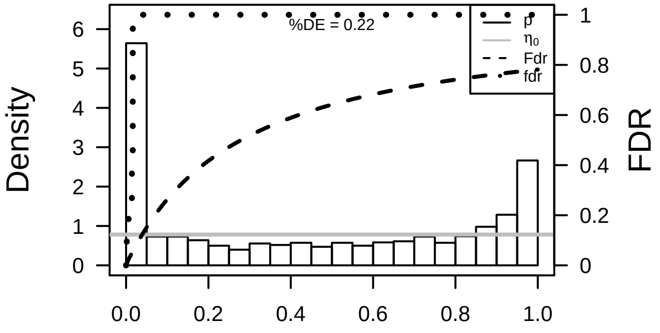

Supplement: Supplementary file 1 [file cells-09-00779-s001.zip › Supplementary materials/FileS4.zip!/FileS3/TFs - Results/Summary Sheets - Samples/Zhongwan.6.25.2..pdf]
